# Cystic Fibrosis: Modern Diagnostic and Therapeutic Advances from Molecular Pathogenesis to Multidisciplinary Management

**DOI:** 10.3390/genes17070743

**Published:** 2026-06-27

**Authors:** Liqin Ke, Lijun Guan, Yiyao Bao, Chao Tang

**Affiliations:** Children’s Hospital, Zhejiang University School of Medicine, National Clinical Research Center for Children and Adolescents’ Health and Diseases, Hangzhou 310052, China; 6513049@zju.edu.cn (L.K.); 6513035@zju.edu.cn (L.G.)

**Keywords:** cystic fibrosis, CFTR, genotype–phenotype correlation, variant interpretation, genomic diagnostics, precision medicine, CFTR modulators

## Abstract

Cystic fibrosis (CF) is an autosomal recessive disorder caused by pathogenic variants in the cystic fibrosis transmembrane conductance regulator (CFTR) gene. It is increasingly understood through a genomics-informed framework linking variant architecture to phenotype, diagnosis, and therapeutic eligibility. This review summarizes current evidence on CFTR structure, variant interpretation, genotype-phenotype heterogeneity, diagnostic workflows, and modern management. We highlight how full-gene sequencing, curated disease-liability databases, functional testing, and organoid-informed theratyping refine diagnosis and treatment selection; how disrupted chloride and bicarbonate transport drives muco-obstructive airway disease and multisystem complications; and why modulator therapy must still be integrated with respiratory, nutritional, endocrine, hepatobiliary, reproductive, and psychosocial care. We also outline unresolved challenges in rare variants, residual organ damage, ancestry-related diagnostic gaps, and mutation-agnostic therapeutic development.

## 1. Introduction

Cystic fibrosis (CF) is one of the best-characterized monogenic disorders in modern medicine, yet its clinical expression is remarkably complex. The disease is caused by pathogenic variants in the cystic fibrosis transmembrane conductance regulator (CFTR) gene, which encodes an epithelial anion channel that regulates chloride and bicarbonate secretion across multiple organs [1,2,3,4]. Defective CFTR function produces dehydrated secretions, abnormal mucus rheology, impaired mucociliary transport, and epithelial surface dysfunction, ultimately creating the biologic conditions for chronic infection, inflammation, and progressive tissue remodeling [2,4,5,6,7].

Historically, CF was viewed mainly as a severe pediatric disease marked by recurrent chest infection, malabsorption, and premature death. That view is no longer sufficient. Earlier diagnosis through newborn screening, better nutritional and respiratory support, and the development of specialized CF centers have extended survival and increased the proportion of adults living with the disease [8,9,10,11]. As survival has improved, the priorities of care have broadened. In addition to preserving lung function, clinicians must now address endocrine, hepatobiliary, reproductive, psychosocial, and transition-related challenges across the lifespan [8,10,12,13,14,15,16,17].

The scientific turning point in CF occurred when the mechanistic understanding of CFTR dysfunction began to translate into therapeutic design. Functional classification of CFTR variants linked molecular defects in synthesis, trafficking, gating, conductance, or stability to candidate pharmacologic strategies, shifting the field from a purely consequence-oriented model to one focused on correction of the basic defect [18,19,20,21,22,23]. This transition made CF an important proof of principle for precision medicine, particularly in respiratory disease.

The arrival of CFTR modulators has further reshaped both expectations and clinical practice. Potentiators and correctors have evolved from mutation-specific interventions to highly effective triple-combination regimens. In many genetically eligible individuals, these therapies improve lung function, reduce pulmonary exacerbations, increase body weight, and improve patient-reported quality of life [23,24,25,26,27,28,29,30,31,32,33,34,35]. Even so, disease burden remains substantial. Many patients enter the modulator era with established bronchiectasis, chronic infection, or extrapulmonary organ damage, whereas others remain ineligible or experience incomplete response, intolerance, or delayed access [10,13,16,20,23,32,33,36,37].

**Figure 1:**
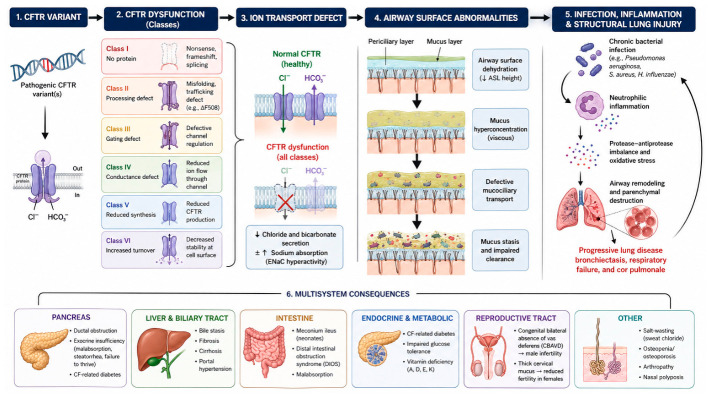
Conceptual genomics-to-phenotype framework for cystic fibrosis, linking CFTR variant architecture to multisystem disease. Legend: The schematic summarizes how different forms of CFTR dysfunction, including defects in synthesis, trafficking, gating, conductance, splicing, or membrane stability, impair chloride and bicarbonate transport and thereby drive airway surface dehydration, mucus hyperconcentration, defective mucociliary transport, chronic infection, inflammation, and progressive structural lung injury. Parallel consequences occur in the pancreas, liver, intestine, endocrine organs, and reproductive tract. The figure is intended as a literature-based conceptual summary rather than a quantitative mutation-frequency map.

A modern review of CF for a genomics-oriented readership must do more than summarize symptoms or therapies. It should explain how CFTR variant architecture, allelic heterogeneity, genotype-phenotype relationships, and population-specific allele distributions shape diagnosis, therapeutic eligibility, and residual uncertainty in daily practice. It should also clarify why genomic information retains clinical meaning only when integrated with physiologic testing, longitudinal phenotype data, and specialist multidisciplinary care. In the following sections, this review links molecular pathogenesis, variant interpretation, diagnostic frameworks, contemporary treatment, and future therapeutic development within a single narrative structure. Figure 1 and Table 1, Table 2, Table 3 and Table 4 are used to organize this genomics-to-clinic framework.

CF remains an instructive model for translational genomics because few monogenic disorders have progressed so visibly from gene discovery to protein-level mechanism, assay development, and mutation-directed treatment. Yet the genomic story is no longer limited to classical mutation classes. Current practice must consider complex alleles, splice-altering and deep intronic variants, large deletions or duplications, modifier background, and ancestry-associated underrepresentation in reference datasets, all of which can alter diagnostic interpretation or modulator eligibility. For this reason, CF is best understood not only as a channelopathy but also as a disease of genomic interpretation.

The epidemiologic profile of CF is also evolving. Although historically considered most common in populations of European ancestry, broader genomic testing and improved awareness have revealed more diverse mutation spectra, founder effects, and geographically variable diagnostic patterns than were once assumed [18,38,39,40,41]. This issue is especially important in Asian and East Asian settings, where diagnosed prevalence is lower, diagnostic delay remains common, and the CFTR variant spectrum differs substantially from that seen in Western cohorts. Chinese series, for example, show fewer classic high-frequency alleles and greater relative representation of rare or atypical variants. This matters because mutation panels and modulator-eligibility frameworks designed around common Western alleles may underperform in underrepresented populations. A contemporary review, therefore has to address not only disease biology but also the implications of diagnostic equity, variant interpretation, and treatment accessibility across different healthcare settings [10,21,22,33,40,41,42].

From a diagnostic perspective, CF has become a moving target because advances in sequencing repeatedly change what counts as an interpretable result. Once expanded newborn screening and broader sequencing are introduced, clinicians must decide how to interpret rare alleles, whether additional copy-number or segregation analysis is required, how to manage screen-positive inconclusive cases, and when physiologic or functional testing should be used to resolve uncertainty [9,11,21,40,41,42,43,44,45]. The genomic era has therefore made diagnosis richer, but not simpler.

This broader framing is especially relevant for review writing because readers now approach CF from very different professional standpoints. A molecular biologist may look for a synthesis of CFTR structure-function relationships and variant interpretation frameworks, while a clinician may need a practical roadmap linking genotyping, sweat chloride interpretation, therapeutic eligibility, and residual disease management [2,4,8,9,10,20,23,36]. A useful contemporary review must therefore connect levels of explanation rather than catalogue facts in isolation.

Taken together, these changes explain why CF is now frequently used as a benchmark for discussing precision medicine and translational genomics. The central challenge is no longer simply to identify a CFTR variant, but to determine how variant architecture, functional consequence, assay evidence, and longitudinal phenotype should be integrated into diagnosis, prognosis, and treatment selection [2,18,19,20,21,22,23,40,41,42,43,44,45,46,47].

Put differently, the contemporary importance of CF lies in its ability to show how modern medicine must connect bench science, clinical evidence, and delivery systems if it wants to change prognosis at scale [2,9,10,23,47].

## 2. Molecular Pathogenesis of Cystic Fibrosis

### 2.1. CFTR Genetics and Functional Heterogeneity

The CFTR gene, located on chromosome 7q31.2, spans 27 exons and encodes a 1480-amino acid membrane protein in the ATP-binding cassette transporter superfamily [18,19]. Unlike most members of that family, CFTR functions primarily as a cyclic AMP-regulated epithelial ion channel [48,49,50]. Its architecture includes two membrane-spanning domains, two nucleotide-binding domains, and a regulatory domain whose phosphorylation and ATP-dependent conformational changes control channel opening and closing [51,52]. Through chloride and bicarbonate transport, CFTR influences surface hydration, luminal pH, mucus expansion, and epithelial defense across multiple organs, including the airways, intestine, pancreas, biliary tree, sweat glands, and reproductive tract [2,5,6,7,18].

CFTR variation is both extensive and clinically consequential. Disease-associated alleles include missense substitutions, nonsense variants, small insertions or deletions, canonical and non-canonical splice variants, deep intronic changes, and promoter or regulatory defects. Less common cases involve exon-level deletions, duplications, or rearrangement-associated events [21,22,39,40,41,42]. Penetrance can differ markedly according to functional effect, allelic context, and modifier background. The traditional mutation-class system remains useful because it organizes variants into biologically meaningful groups such as absent protein synthesis, trafficking failure, gating dysfunction, impaired conductance, reduced protein abundance, and decreased membrane stability. Still, real-world genotypes are often more complex than this framework suggests [18,19,21,22].

The shift from mutation naming to variant interpretation is especially important in modern practice. Large curated resources, disease-liability analyses, full-gene sequencing, and copy-number-aware workflows now support a more precise distinction between clearly pathogenic alleles, variants of variable clinical consequence, and incidental or low-penetrance findings [21,22,40,41,42]. This distinction affects diagnosis, drug eligibility, counseling, cascade testing, and the design of mutation-specific or mutation-agnostic clinical trials. Table 1 summarizes the main CFTR variant classes together with the interpretive caveats most relevant to a genomics-focused readership.

**Table 1:** Genomically relevant classes and interpretive features of CFTR variants.

Variant Class/Architecture	Representative Variants or Mechanisms	Predominant Molecular Consequence	Genotype-Phenotype/Interpretive Caveats	Therapeutic or Diagnostic Relevance
I	G542X, W1282X; nonsense, frameshift, or some exon-disrupting rearrangements	Absent or markedly reduced full-length protein synthesis	Usually associated with classic CF, but severity may still be modified by cis/trans context and background genes	Often modulator-ineligible; supports interest in RNA-, read-through, or gene-based strategies [18,19,21,22,40]
II	F508del, N1303K; misfolding and trafficking variants	Defective processing and reduced cell-surface delivery	Common severe phenotype, but response varies with second allele and complex-allele context	Corrector-based combinations and triple therapy for eligible genotypes [18,19,21,23,24,25,26,40]
III	G551D, S549N	Defective gating with residual surface expression	Can show substantial pharmacologic responsiveness despite severe baseline dysfunction	Potentiator-responsive archetype [18,19,21,27,40]
IV	R117H, D1152H	Reduced channel conductance	Penetrance and organ involvement are variable; interpretation often requires phenotype integration	Variable modulator response; caution with isolated genotype-based prediction [18,19,21,41,42]
V	3849 + 10kbC>T, 2789 + 5G>A; promoter- or splice-reducing variants	Reduced transcript abundance or abnormal splicing	Residual function and disease expression are heterogeneous; deep intronic events may be missed by limited panels	Some variants are treatment-responsive; may require expanded sequencing or transcript-aware interpretation [19,21,39,40,41,42]
VI	Membrane-unstable variants; complex alleles with cis-modifying effects	Reduced surface stability or combined multi-step dysfunction	Class labels can oversimplify variants affected by cis context, structural complexity, or assay platform	May need combined functional testing, organoid assays, and individualized therapeutic decisions [21,22,40,42,53]

At a mechanistic level, CFTR serves as more than a passive chloride pore. Its bicarbonate transport capacity is increasingly recognized as central to epithelial surface chemistry, particularly the pH environment that influences antimicrobial activity, mucin unfolding, and secretion viscosity [2,5,6,7]. In practical terms, this means that variant effects cannot be judged only by whether chloride transport is reduced; the broader impact on airway surface physiology may differ depending on whether a variant primarily impairs synthesis, trafficking, splicing, gating, or membrane stability. This nuance helps explain why variants with apparently similar residual function can still be associated with different clinical patterns and different responses to pharmacologic modulation [19,21,22,40].

The mutation-class framework is most valuable when treated as a translational tool rather than an absolute taxonomy. Many disease-causing alleles exert more than one molecular effect, and some variants behave differently depending on cell type, cis modifiers, interacting polymorphisms, or assay platform [21,22,41,42]. Clinical interpretation therefore, increasingly relies on convergent evidence: population frequency data, disease penetrance estimates, in vitro functional assays, patient-derived organoid response patterns, and observed clinical phenotypes [22,40,41,42]. The maturation of this evidence ecosystem has practical consequences for patient care because it refines which individuals qualify for existing therapies, which remain candidates for compassionate-use pathways, and which are most relevant for future genomically stratified studies.

Another important theme in CFTR biology is that functional consequence does not always align perfectly with classical disease-severity categories. Some variants are associated with pancreatic sufficiency, isolated congenital bilateral absence of the vas deferens, or milder respiratory trajectories, yet can still produce clinically significant disease in the presence of modifying genetic or environmental factors [18,21,22,41,42]. This creates a continuum rather than a sharp boundary between classic CF, atypical CF, and CFTR-related disorders. For clinicians and investigators, that continuum has practical implications for diagnosis, family counseling, registry inclusion, and therapeutic prioritization.

The increasing sophistication of CFTR interpretation also has important implications for population screening and personalized medicine. Variant panels built around common disease-causing alleles remain useful in high-prevalence populations, but they may perform inadequately where mutation spectra are more heterogeneous or where underrepresented populations have historically been missed in reference databases [38,39,40,41]. Broader sequencing strategies partially address that problem, yet they also increase the burden of interpreting rare alleles, structurally complex events, and ancestry-specific findings whose clinical effect may only become clear through collaborative data sharing and ongoing phenotype collection. In this respect, CFTR genetics has become a model for precision medicine in the strongest sense: the laboratory result acquires meaning only when anchored to curated databases, functional evidence, clinical phenotype, and longitudinal follow-up.

For reviewers and readers, one practical implication is that any discussion of mutation class should be paired with a reminder that therapeutic relevance is contextual. A variant may be categorized in one way at the molecular level yet still show clinically meaningful variability in phenotype or drug response because of modifier effects, assay differences, phase uncertainty, or allelic complexity [21,22,40,42,54]. The strongest contemporary literature therefore, favors an integrated approach in which classification supports, but does not replace, individualized interpretation.

This is why genotype interpretation should be viewed as a living process informed by accumulating data, not as a fixed classification completed once and for all at the time of diagnosis [40,41,42].

### 2.2. From Epithelial Ion Transport Failure to Multisystem Disease

The central pathogenic consequence of CFTR dysfunction is defective epithelial ion and fluid handling. In the airways, reduced chloride and bicarbonate secretion contributes to airway surface liquid depletion, altered mucus hydration, abnormal mucin unfolding, and impaired ciliary transport [5,6,7]. These changes precede overt bronchiectasis and help explain why early CF lung disease can progress even when symptoms remain subtle. Once mucus retention is established, stagnant secretions create an environment favorable to microbial persistence and sustained innate immune activation [5,7,55,56].

Chronic airway infection is not merely a secondary event but a major amplifier of disease progression. Pathogens such as Pseudomonas aeruginosa adapt to the CF airway through biofilm formation, quorum-sensing shifts, metabolic specialization, and antibiotic tolerance [55,56,57]. Neutrophil-dominant inflammation, protease excess, oxidative stress, and epithelial injury then reinforce a self-perpetuating cycle in which infection worsens obstruction and obstruction further stabilizes infection. Over time, this cycle drives airway remodeling, bronchiectasis, and irreversible lung-function loss [1,4,5,7,56].

The same secretory defect helps explain the broader multisystem phenotype of CF. In the pancreas, thickened secretions obstruct small ducts and damage acinar tissue, leading to exocrine pancreatic insufficiency and malabsorption [2,4,58]. In the liver and biliary tract, impaired ductal secretion contributes to cholestatic injury and risk of progressive hepatobiliary disease [16]. In the intestine, reduced luminal hydration promotes meconium ileus in infancy and distal intestinal obstructive syndrome later in life [2,4,8]. Additional consequences include cystic fibrosis-related diabetes (CFRD), low bone mineral density, chronic rhinosinusitis, and reproductive tract abnormalities [12,13,37,59,60].

Importantly, not all patients traverse the same biologic pathway to the same extent. Modifier genes, environmental exposures, treatment adherence, socioeconomic context, and pathogen ecology contribute to genotype-phenotype variability [53,54]. This is one reason why patients sharing a major genotype can still exhibit different ages at presentation, pancreatic status, infection burden, and rates of pulmonary decline. Figure 1 illustrates the mechanistic trajectory from CFTR dysfunction to multisystem disease, while the sections below show how this biology informs diagnosis and management.

The concept of muco-obstructive disease has become central to modern understanding of CF because it links a microscopic defect in epithelial ion transport with the macroscopic pattern of progressive airway injury visible on imaging and pathology [5,6,7]. Hyperconcentrated mucus increases cohesive and adhesive forces, slows ciliary transport, and favors the retention of inflammatory material within the small airways [61,62,63]. Once this state is established, pathogen exposure, epithelial stress, and immune-cell recruitment interact in a nonlinear way, so that even modest infectious or inflammatory insults may trigger disproportionate downstream damage [5,7,55,56]. This helps explain why some patients show radiographic and microbiologic progression even during periods of apparently limited clinical symptom burden.

Extrapulmonary pathogenesis follows the same general logic of impaired secretion, obstruction, and secondary tissue injury, but each organ expresses this logic differently [64,65,66]. Pancreatic injury often begins early and can be largely irreversible once ductal obstruction and acinar loss are established, which is why pancreatic status correlates strongly with genotype severity [2,18,58]. Hepatobiliary disease is more heterogeneous and likely reflects a combination of altered ductal secretion, bile composition, local inflammation, and host-specific susceptibility factors [16]. CFRD likewise emerges not from a single metabolic lesion but from the cumulative effect of pancreatic damage, reduced insulin reserve, infection burden, inflammation, and evolving body-composition changes over time [13,59].

The immunobiology of CF lung disease is also more complex than a simple model of “infection causing inflammation.” Defective epithelial surfaces may alter host defense even before chronic pathogen colonization becomes established, and once inflammation is triggered, it can persist through dysregulated neutrophil recruitment, protease-antiprotease imbalance, extracellular DNA release, oxidative stress, and altered innate immune signaling [5,6,7,55,56]. As a result, airway damage can progress through intertwined infectious and sterile inflammatory pathways. This distinction is clinically relevant because inflammatory activity may persist despite partial reduction in microbial burden, helping to explain residual lung disease in some patients after modulator therapy.

An additional reason the pathogenesis of CF remains clinically important, even in the era of highly effective modulators, is that it explains the persistence of residual disease after major pharmacologic improvement [67]. Once bronchiectasis, microbial adaptation, pancreatic fibrosis, or chronic hepatobiliary injury has been established, restoration of partial CFTR function may halt further injury more effectively than it reverses damage that is already structurally fixed [5,7,16,20,23,32,33]. This distinction between preventing progression and reversing established injury has practical implications for how clinicians counsel families, interpret trial results, and decide how aggressively to maintain supportive care after starting modulators. It also reinforces the value of early diagnosis and early disease-modifying intervention: the same therapy may have very different long-term consequences depending on whether it is introduced before or after chronic tissue remodeling is entrenched. In this sense, pathogenesis is not merely a background science section; it defines the biologic window in which treatment is most likely to alter life-course outcomes.

The pathogenesis section is also a reminder that timing matters biologically. Once the cycle of obstruction, infection, inflammation, and remodeling becomes self-sustaining, treatment has to work against both the initiating CFTR defect and the downstream tissue consequences of years of disease activity [5,7,55,56]. This helps explain why early-life intervention is so central to current CF strategy and why residual disease persists in many patients despite access to better pharmacology than any previous generation had available.

The practical lesson is straightforward: the more completely clinicians understand this cascade, the better they can judge which manifestations are likely to be reversible, partially reversible, or primarily preventable with earlier intervention [5,6,7,56].

## 3. Clinical Spectrum and Diagnostic Evaluation

### 3.1. Multisystem Clinical Manifestations Across the Lifespan

The clinical phenotype of CF is highly heterogeneous, but respiratory disease remains the dominant determinant of long-term outcome. Early manifestations may include chronic cough, recurrent wheeze, prolonged recovery from respiratory infections, exercise intolerance, and persistent sputum retention, although substantial structural disease can be present even before symptoms become dramatic [7,8,9]. Recurrent pulmonary exacerbations, chronic endobronchial infection, and progressive bronchiectasis often define the transition from early childhood disease to more established pulmonary impairment [2,4,5,55,56].

Extrapulmonary manifestations are equally important for prognosis and quality of life. Pancreatic insufficiency contributes to steatorrhea, poor growth, micronutrient deficiency, and reduced lean body mass, all of which can worsen pulmonary resilience [8,58]. Hepatobiliary disease may remain subclinical for long periods before presenting with laboratory abnormalities, splenomegaly, portal-hypertensive findings, or clinically meaningful fibrosis [16]. Gastrointestinal symptoms, abdominal pain, constipation, and distal intestinal obstruction further increase treatment burden and can reduce nutritional stability [2,8,16,58].

Endocrine and systemic complications become increasingly prominent with age. CFRD is a major comorbidity associated with worse nutrition and pulmonary health, and its pathophysiology differs from both classic type 1 and type 2 diabetes because progressive pancreatic damage and illness-related insulin insufficiency coexist [13,59]. Bone disease, delayed puberty, chronic rhinosinusitis, reduced fertility, and pregnancy-management complexity all add to the cumulative burden of adult CF care [12,37,60]. Table 2 provides a concise overview of the major manifestations and the assessment domains that should be reviewed longitudinally.

**Table 2:** Major respiratory and extrapulmonary manifestations of cystic fibrosis and key clinical assessment points.

Organ System	Representative Manifestations	Core Assessment Tools	Clinical Implications
Lower airways	Chronic cough, sputum retention, bronchiectasis, recurrent exacerbations	Spirometry, sputum microbiology, imaging, symptom review	Principal driver of long-term morbidity and mortality [2,4,5,7,55,56,57]
Pancreas and nutrition	Exocrine pancreatic insufficiency, steatorrhea, poor growth, vitamin deficiency	Growth trajectory, fecal elastase, dietary assessment, vitamin monitoring	Directly affects pulmonary resilience and development [8,58]
Liver and biliary tract	Cholestasis, fibrosis, portal hypertension, CF-associated liver disease	Liver enzymes, ultrasound-based surveillance, specialist hepatology review	Requires risk-stratified longitudinal monitoring [16]
Endocrine and bone	CFRD, low bone mineral density, fractures	Glucose screening, HbA1c context review, bone density assessment	Linked to worse nutrition, lung health, and quality of life [13,37,59]
Upper airways	Chronic rhinosinusitis, nasal polyposis	ENT evaluation and symptom-directed imaging	Adds to treatment burden and airway symptom load [60]
Reproductive health	Male infertility, subfertility, pregnancy-management complexity	Fertility counseling, medication review, multidisciplinary obstetric planning	Increasingly relevant in the adult CF population [12,60]

The natural history of CF is also shaped by treatment era. In the post-modulator setting, some patients present with milder respiratory symptoms yet still require close surveillance for residual structural lung disease, chronic infection, obesity or altered body composition, and persistent extrapulmonary complications [10,23,32,33]. For this reason, clinical evaluation should remain multidimensional even when apparent pulmonary stability improves.

Age modifies not only the severity of CF manifestations but also the way they present. In infancy and early childhood, the most important clues may be meconium ileus, poor weight gain, salt-loss syndromes, recurrent cough, or the consequence of a positive newborn screen rather than overt chronic sputum disease [9,11,43,46]. In adolescence and adulthood, by contrast, patients may present with chronic sinus disease, exercise limitation, recurrent exacerbations, CFRD, infertility, or complex treatment-adherence challenges superimposed on established bronchiectasis [8,12,13,14,17,60]. A lifespan perspective is therefore necessary not only for management but also for recognizing how the disease phenotype evolves over time.

The growing adult CF population has also changed the interpretation of “disease burden.” Earlier eras emphasized survival itself, whereas contemporary care increasingly addresses symptom control, participation in education or employment, reproductive decision-making, body image, mood, treatment fatigue, and the impact of long-term therapy on daily life [10,12,14,15,17,68]. Consequently, clinical assessment should include patient-reported outcomes, quality-of-life measures, and psychosocial review rather than relying only on spirometry, microbiology, or weight. This broader framing is especially important in patients receiving modulators, where improvements in lung function may coexist with persistent fatigue, mental-health strain, chronic infection, or significant extrapulmonary disease.

Clinicians should also recognize that manifestations may be uncoupled across organ systems. A patient with relatively preserved lung function may nevertheless have significant sinus disease, nutritional challenge, diabetes risk, or psychosocial burden, while another with marked bronchiectasis may retain better pancreatic function than expected for genotype [8,13,15,54,58,60]. This decoupling reinforces the need for review templates that are multisystem by design. It is not enough to ask whether a patient is coughing less or whether the most recent FEV1 has improved; good care requires active surveillance for organ-specific problems that may progress outside the clinician’s immediate field of focus.

The clinical burden of CF should therefore be conceptualized in layered rather than isolated terms. Pulmonary symptoms affect exercise tolerance, school attendance, sleep quality, and work capacity; malabsorption affects growth, bone health, and immune resilience; CFRD influences body composition and pulmonary prognosis; chronic rhinosinusitis and reproductive concerns shape daily quality of life and long-term planning [8,12,13,37,58,60]. When these burdens cluster, they can create a cumulative effect far greater than any single complication considered alone. This clustering is especially important in adolescence and adulthood, where the day-to-day experience of disease often includes heavy self-management requirements, uncertainty about future progression, and the need to integrate treatment with ordinary life milestones. For that reason, a comprehensive review should avoid presenting extrapulmonary disease as a set of secondary complications. In current practice, it is more accurate to describe CF as a multisystem syndrome with pulmonary predominance, in which organ-specific problems continually interact with one another and with the practical burden of care.

Another clinical challenge is that patient-reported burden may not mirror clinician-defined severity. Some individuals normalize chronic symptoms because they have never experienced a truly asymptomatic baseline, while others may be highly distressed by complications that receive less attention in routine pulmonary review, such as sinus disease, gastrointestinal discomfort, or reproductive uncertainty [12,15,60,68]. Expanding the clinical lens, therefore, improves not only detection of complications but also the alignment between medical priorities and lived patient experience.

A broad clinical review is therefore not a matter of completeness for its own sake; it is the most accurate reflection of how patients actually experience CF over time [8,12,13,15].

### 3.2. Diagnostic Pathways, Baseline Evaluation, and Prognostic Stratification

Contemporary diagnosis of CF is anchored in the demonstration of CFTR dysfunction together with a compatible clinical or screening context. In many healthcare systems, the pathway begins with IRT-based newborn screening, followed by confirmatory sweat chloride testing and CFTR genotyping in infants with positive screens [9,11,43,46]. Consensus guidelines have strengthened the standardization of this approach, particularly in the interpretation of screen-positive infants, intermediate sweat chloride values, and so-called CFTR-related metabolic syndrome or CF screen positive, inconclusive diagnosis (CRMS/CFSPID) [9,11,43,44].

Sweat chloride remains the most established diagnostic biomarker because it reflects CFTR dysfunction directly and also remains informative during modulator treatment [9,26,27,45]. Modern diagnostic work, however, often extends beyond targeted genotyping. When only one pathogenic variant is identified, or when phenotype and routine results are discordant, additional steps may be needed. These can include full-gene CFTR sequencing with copy-number analysis, review for splice-altering and deep intronic variants, segregation studies, and, in selected cases, physiologic assays such as nasal potential difference or intestinal current measurement [21,22,39,40,41,42,45].

Baseline evaluation after diagnosis should be comprehensive because prognosis is determined by more than genotype alone. Initial assessment should define respiratory burden, microbiology, nutritional status, pancreatic status, hepatobiliary involvement, endocrine risk, and psychosocial needs [8,9,13,15,16,45]. For clinically stable patients, repeated longitudinal measurements often have greater prognostic value than a single cross-sectional estimate because they reveal the tempo of decline, therapeutic response, and adherence barriers over time [8,54]. In addition to spirometry, emerging adjunctive tools such as lung clearance index (LCI), quantitative chest imaging, and selected inflammatory biomarkers can help detect early small-airway disease, define residual structural burden, and monitor therapeutic response in patients whose conventional lung function appears relatively stable [10,45,46,69].

Prognostic stratification in CF is therefore best understood as a layered process. Genotype, variant penetrance, modifier genes, microbiologic profile, nutritional trajectory, and access to highly effective therapy all contribute to risk [32,33,42,54]. Table 3 summarizes the strengths and limitations of the principal tools currently used in diagnosis and baseline evaluation.

**Table 3:** Current screening and diagnostic tools for cystic fibrosis: clinical role, strengths, and limitations.

Diagnostic Modality	Primary Role	Strengths	Limitations
IRT-based newborn screening	Early population-level case detection	Identifies affected infants before overt clinical decline	Requires confirmatory testing; algorithms differ by region [9,11,43,46]
Sweat chloride testing	Core confirmatory test for CFTR dysfunction	Widely accepted, guideline-based, and useful for pharmacodynamic follow-up	Intermediate values require further interpretation [9,43,44,45]
CFTR genotyping, full-gene sequencing, and copy-number analysis	Etiologic confirmation, variant interpretation, and therapeutic eligibility review	Clarifies pathogenic variants, detects broader allelic diversity, and supports precision therapy	Interpretation can remain uncertain in atypical disease, rare alleles, or incompletely represented populations [39,40,41,42,45]
Physiologic and selected functional assays	Problem-solving in unresolved or inconclusive cases	Provides direct evidence of CFTR dysfunction when genomic findings are insufficiently decisive	Limited availability and specialist expertise required [39,41,45]
Functional, imaging, and biomarker assessment	Baseline severity, early disease detection, and response monitoring	Can integrate spirometry, LCI, quantitative imaging, and selected inflammatory markers	Standardization, age dependence, and inter-center availability remain variable [10,45,46,69]

Diagnostic strategy in CF increasingly reflects a balance between sensitivity and interpretive precision. Expanded newborn screening and broader sequencing can identify more infants and atypical cases earlier than before. They also generate more uncertain results, including rare alleles, phase ambiguity, and ancestry-related interpretation gaps [32,33,42,54]. A practical modern framework is therefore stepwise: screening context, sweat chloride testing, full-gene variant analysis with deletion or duplication calling when needed, family-based phasing or segregation, and functional confirmation in unresolved cases. Longitudinal monitoring is also becoming more nuanced, with increasing interest in LCI, quantitative imaging, and inflammatory biomarkers as complements to spirometry when early or residual disease is difficult to capture [10,45,46,69].

Baseline evaluation after diagnosis should establish an organ-specific map of disease burden and a plan for longitudinal surveillance. Early review should cover respiratory history, sputum culture profile, spirometry, imaging context, nutritional assessment, fecal elastase or pancreatic status, liver review, glucose screening strategy, bone-health risk, sinonasal symptoms, reproductive concerns, and psychosocial status [8,12,13,15,16,37,58,60]. Prognosis is then shaped by the interaction between baseline burden and the speed with which effective therapy can be deployed. This is particularly relevant in the modulator era, where patients with similar genotypes may still follow very different long-term trajectories.

Diagnostic uncertainty remains one of the most practically challenging areas of CF medicine. Borderline sweat chloride values, variants of uncertain significance, and phenotypes that do not fit classical expectations can create prolonged ambiguity for families and clinicians [9,44,45]. In these settings, the goal is not simply to attach or withhold a label as quickly as possible, but to establish a monitoring and reassessment framework proportionate to the level of uncertainty. Such an approach acknowledges that some individuals will declare a more definite phenotype over time, while others may remain within an intermediate zone that still warrants specialist follow-up. This longitudinal diagnostic model is increasingly important in the genomic era, where the amount of detectable variation has outpaced the ease of clinical interpretation.

Prognostic stratification in CF is similarly shifting from static categorization toward dynamic surveillance. Historically, clinicians often relied heavily on genotype severity, pancreatic status, or baseline spirometry to estimate risk. Those variables remain useful, but their predictive meaning is increasingly modified by early screening, access to modulators, real-world adherence, socioeconomic context, and the evolving microbiologic environment [8,10,32,33,45,54]. A patient diagnosed through newborn screening and started on effective therapy in early life may follow a dramatically different trajectory from an older patient with the same genotype and longstanding bronchiectasis. Modern prognostic thinking, therefore, places greater emphasis on tempo across lung function, LCI, imaging, microbiology, and treatment tolerance than on a single severity label alone.

Diagnostic refinement also shapes therapeutic opportunity. Accurate characterization of CFTR variants, sweat chloride phenotype, and organ involvement can determine whether a patient receives a currently approved modulator, qualifies for additional functional testing, or should be considered for emerging trial pathways [9,40,41,44,45]. In that sense, diagnosis in modern CF is no longer just about naming a disease; it is about opening or narrowing future care options. That is one reason why diagnostic quality has become even more important in the era of precision treatment.

In everyday practice, high-quality diagnosis and follow-up planning are often the first steps that determine whether later therapeutic opportunities can be used to full advantage [9,11,44,45].

## 4. Contemporary Therapeutic Strategies and Comprehensive Supportive Management

### 4.1. CFTR Modulator Therapy and Respiratory Management

#### 4.1.1. Precision Pharmacotherapy in the Modulator Era

CFTR modulators constitute the most significant therapeutic advance in the history of CF. Ivacaftor demonstrated that restoration of channel gating could produce clinically meaningful improvements in patients with responsive variants [27]. Subsequent combinations of correctors and potentiators broadened treatment toward F508del-related disease, with lumacaftor/ivacaftor and tezacaftor/ivacaftor establishing proof of progressive correction of trafficking defects [28,29,30]. Triple-combination regimens then raised the therapeutic ceiling considerably by improving both protein processing and channel function, leading to larger gains in lung function and symptom control [24,25,26,31,34,35].

The impact of modulator therapy extends beyond spirometry. Clinical trials and real-world studies show reductions in exacerbation frequency, improved nutritional status, lower sweat chloride values, and better patient-reported respiratory status [10,23,24,26,31,32,33,34]. Extrapulmonary effects are also increasingly relevant. Improvements in sinus symptoms, weight trajectory, treatment burden, and some domains of reproductive or endocrine planning may occur, but these gains are not uniform and do not eliminate the need for ongoing multisystem surveillance. In practice, follow-up is therefore becoming more multidimensional, incorporating extrapulmonary outcomes, microbiologic trajectory, and adjunctive markers of treatment response.

Despite their transformative effect, modulators should not be presented as universally curative. Structural bronchiectasis, chronic airway infection, residual inflammation, nonresponsive variants, intolerance, and inequitable access remain substantial limitations [10,20,23,32,40]. Clinicians must therefore balance enthusiasm for precision pharmacology with realistic recognition that response varies and that many patients still require intensive supportive care. This is especially important for patients with nonsense variants, severely disruptive alleles, unresolved therapeutic eligibility, or rare genotypes for which approved options remain absent or incomplete.

The progression from ivacaftor to highly effective triple therapy illustrates a broader principle in CF pharmacology: restoration of clinically meaningful CFTR function often requires addressing several molecular bottlenecks at once [24,25,26,27,28,29,30]. For many F508del-containing genotypes, correction of trafficking alone is insufficient because rescued protein may still display defective channel opening or reduced stability at the membrane [70,71]. Combination therapy, therefore, became necessary not simply to increase efficacy incrementally, but to approximate a more complete biologic rescue. This pharmacologic logic has helped define the current era of CF care and continues to shape development pipelines for newer regimens [20,23,24,31,34,35].

Real-world use has also shown that the clinical meaning of “response” is multidimensional. Improvement may be seen in lung function, exacerbation frequency, weight gain, sweat chloride, exercise tolerance, sinus symptoms, treatment burden, or subjective well-being, and these domains do not always change in parallel [10,23,32,33]. Some patients show striking symptomatic relief with only modest spirometric change, whereas others achieve biochemical improvement but continue to experience chronic infection or extrapulmonary complications. This heterogeneity reinforces the need for structured post-initiation monitoring rather than a simplistic binary classification of responder versus non-responder.

There is also a broader conceptual lesson in modulator therapy: effective treatment is not synonymous with complete biologic normalization. Even when CFTR function improves substantially, prior structural damage, airway colonization history, nutritional deficits, and entrenched care patterns can continue to shape the patient’s trajectory [10,23,26,32]. This is why studies of long-term outcomes and registry data remain so important. The key question is no longer only whether a regimen improves short-term lung function or sweat chloride, but whether it changes the cumulative burden of disease over decades, delays transplant, preserves fertility and endocrine health, mitigates hepatobiliary complications, or reduces late adult morbidity.

Precision pharmacotherapy has also altered the scientific questions surrounding clinical trials. Early landmark studies were designed to show that modulating a defective protein could improve clinical outcomes at all [27,28,29,30]. Current and future studies ask more focused questions: which combinations deliver the most durable rescue, which biomarkers best capture response, whether therapy can be simplified without loss of efficacy, how early treatment changes life-course outcomes, and how rare variants can be evaluated when large randomized trials are impractical [20,23,24,31,32,33,34,35]. This shift has methodological consequences. Registry-based evidence, translational assay data, and longitudinal cohort analysis are becoming more important complements to conventional randomized trials. Treatment discussion should therefore integrate efficacy, eligibility, implementation, and evidence hierarchy rather than merely list approved drugs.

The practical consequence for clinicians is that modulator review should be longitudinal and multidimensional. Initiation decisions, CYP3A-mediated drug–drug interaction review, liver biochemistry surveillance, symptom reassessment, microbiology review, and discussion of treatment expectations all need to be revisited over time rather than completed once at prescription [10,23,26,32,33,34]. Some patients may also require explicit discussion of mood, sleep, or neuropsychiatric symptoms, particularly when subjective tolerability does not mirror the magnitude of physiologic benefit. Structured follow-up is also critical for individuals with rare or incompletely responsive variants, who may need repeated reassessment of theratyping results, compassionate-use pathways, or trial eligibility.

For that reason, discussions of modulators should consistently include both their transformative benefits and the patients who remain only partially served by the current pharmacologic era. Modulator-ineligible individuals still depend on careful airway care, infection control, nutritional and endocrine support, and access to emerging mutation-agnostic strategies. A balanced review must therefore discuss not only efficacy in eligible populations, but also the clinical reality of residual disease, incomplete response, and persistent therapeutic exclusion.

#### 4.1.2. Airway Clearance, Infection Control, and Advanced Pulmonary Care

Respiratory care remains essential even in the modulator era because mucus retention, chronic infection, and pre-existing structural lung damage often persist [8,23]. Airway clearance techniques, exercise, hypertonic saline, dornase alfa, and individualized physiotherapy plans continue to form the backbone of daily management for many patients. These therapies remain particularly relevant when bronchiectasis, lobar mucus retention, or fluctuating symptom burden persists despite modulator treatment. The exact regimen should be adapted to age, treatment burden, symptom pattern, and response to modulators rather than applied rigidly.

Infection management requires equal attention. Regular microbiologic surveillance, prompt treatment of pulmonary exacerbations, early eradication strategies for new Pseudomonas acquisition, and infection-prevention procedures within CF programs remain core elements of respiratory care [55,56,57,72]. Chronic suppressive inhaled or systemic antibiotic strategies may still be needed in selected patients with persistent pathogen burden. These measures are especially important because modulator therapy, although beneficial, does not reliably eliminate established airway pathogens or fully reverse bronchiectatic architecture.

Advanced pulmonary care should also be integrated proactively rather than deferred until a crisis develops. Patients with persistent severe lung disease may require oxygen therapy, ventilatory support in selected circumstances, palliative symptom planning, and timely consideration of lung transplantation [36]. Referral for transplant is most effective when it occurs before irreversible end-stage decline compresses the decision window, and modern criteria should be interpreted in the context of residual disease burden rather than a simplistic assumption that modulator therapy has removed the need for transplant planning.

One of the central clinical debates in the modulator era is whether conventional respiratory therapies can or should be reduced after substantial clinical improvement. At present, the answer is individualized rather than universal. Many patients experience less sputum production, fewer exacerbations, and improved daily function on modulator therapy, but evidence remains insufficient to assume that airway clearance, mucolytics, or surveillance microbiology can be safely withdrawn wholesale in all cases [8,23,32,33]. Reduction strategies, when considered, should be guided by disease severity, prior bronchiectasis burden, chronic infection profile, and the patient’s demonstrated stability over time.

Infection prevention remains particularly important because the epidemiology of CF airway infection may change without becoming clinically irrelevant. Chronic Pseudomonas aeruginosa, Burkholderia cepacia complex, nontuberculous mycobacteria, and fungal colonization can all complicate the course of disease, even when overt symptoms improve [55,56,57,72]. Long-term antibiotic exposure also raises concerns about antimicrobial resistance, multidrug-resistant organisms, and the narrowing of future treatment options. Program-level infection control, timely culture review, and rational antibiotic stewardship, therefore, remain essential parts of care.

Advanced pulmonary management should likewise be viewed across a trajectory rather than at a single threshold. Patients may move gradually from predominantly outpatient care toward greater dependence on intravenous antibiotics, oxygen, ventilatory support, or transplant discussion, with long periods of partial stability in between [36,57]. Recognizing these transitions early allows teams to plan more effectively for symptom relief, family counseling, work or education disruption, and evaluation at transplant-capable centers. In this sense, the best advanced CF care is anticipatory care: it reduces crisis-driven decisions and gives patients more opportunity to participate meaningfully in major therapeutic choices.

A further challenge in respiratory management is that improved symptoms can sometimes obscure ongoing risk. A patient who coughs less after modulator initiation may still carry significant bronchiectasis, persistent mucus retention in specific lobes, chronic bacterial colonization, or a history of severe exacerbations that continues to predict future instability [23,36,55,56,57]. For this reason, decisions about de-escalating therapy should ideally be made within a structured framework that incorporates microbiology, imaging history, prior exacerbation frequency, symptom burden, and patient preference.

This is why respiratory management in CF remains an exercise in careful adjustment rather than wholesale replacement. New therapies have shifted the baseline, but they have not erased the need for microbiologic vigilance, individualized airway clearance, and advanced planning in severe disease [36,55,56,57]. In practice, the most successful programs are those that treat the modulator era as an opportunity to refine respiratory care, not abandon it.

Another unresolved issue in the post-modulator era is residual neutrophilic airway inflammation. Mutation-agnostic anti-inflammatory strategies, therefore, remain of interest. One example is the oral DPP1 inhibitor brensocatib, which reduces activation of neutrophil serine proteases and has lowered exacerbation burden in non-CF bronchiectasis trials [69]. At present, however, this evidence should be interpreted cautiously in CF. Brensocatib has not yet been established as a standard CF therapy, and any discussion of its role should emphasize that the most robust efficacy data derive from bronchiectasis outside the CF population.

### 4.2. Nutritional, Metabolic, and Organ-Specific Supportive Care

#### 4.2.1. Pancreatic, Endocrine, and Bone Health Management

Nutritional management is not adjunctive in CF; it is foundational. Growth failure, malnutrition, and reduced lean mass correlate with worse pulmonary outcomes, and aggressive nutrition support has long been a major reason survival improved before modulators became available [8,58]. Pancreatic enzyme replacement therapy, liberal but individualized dietary planning, monitoring of fat-soluble vitamins, and escalation to supplemental enteral nutrition when required remain central to the management of pancreatic insufficiency [8,58].

The post-modulator era has not removed the need for metabolic surveillance; it has made that surveillance more nuanced. Some patients continue to struggle with malabsorption and low weight, while others experience rapid weight gain, altered appetite, or changing body composition after modulator initiation [10,23,32,33]. Consequently, nutritional care should be dynamic and revisit caloric prescriptions, micronutrient goals, and exercise planning over time rather than assuming static energy needs.

Endocrine and skeletal complications require equally structured management. Current practice continues to favor regular OGTT-based screening for CFRD from late childhood onward, with insulin remaining the standard treatment once clinically meaningful dysglycemia is established [13,59]. Bone disease screening and intervention are likewise important, particularly in patients with delayed puberty, glucocorticoid exposure, chronic inflammation, vitamin D deficiency, or a history of fractures [37].

Nutritional care in CF has become more sophisticated because the aims of treatment are no longer limited to avoiding frank malnutrition. Clinicians increasingly focus on body composition, muscle preservation, exercise capacity, and the metabolic consequences of both chronic inflammation and successful modulator therapy [8,10,33,58]. This broader approach is important because traditional weight-centered metrics may miss patients who remain sarcopenic, metabolically vulnerable, or at risk of functional decline despite apparent improvement in body mass index.

CFRD and bone disease exemplify the need for proactive, supportive medicine. Both may progress quietly, both materially influence respiratory health and quality of life, and both are often underappreciated when clinical attention is dominated by pulmonary issues [13,37,59]. In the post-modulator era, it is increasingly important to define how far highly effective therapy modifies glucose tolerance, insulin requirement, bone turnover, and long-term metabolic outcomes. Until those questions are answered more clearly, routine screening and timely intervention should remain integral rather than optional components of care.

Another important clinical issue is that supportive metabolic care may need recalibration as therapeutic success changes the phenotype of the disease. Historically, undernutrition dominated the nutritional conversation in CF, but some patients receiving highly effective modulators now face challenges related to overweight, altered insulin sensitivity, or shifts in lifestyle and exercise behavior [10,13,32,33,58]. This does not diminish the importance of classical nutritional support; rather, it means that CF dietetics and endocrine care must become more individualized and less anchored to assumptions derived entirely from the pre-modulator era.

Supportive metabolic care is also where some of the most important unanswered post-modulator questions now reside. It remains uncertain how fully highly effective therapy modifies pancreatic exocrine function after infancy, whether longstanding micronutrient deficits normalize over time, how CFRD epidemiology will shift in adults treated earlier in life, and whether bone outcomes will improve automatically or still require intensive adjunctive management [13,32,37,58,59]. These uncertainties should not be mistaken for evidence that supportive care has become less important. On the contrary, they reinforce the need for structured monitoring so that new phenotypes are recognized rather than assumed away.

For the same reason, metabolic care should remain embedded in every major follow-up framework. Weight trajectory, glucose tolerance, pancreatic replacement efficacy, micronutrient status, and bone-health surveillance all influence long-term resilience and functional capacity [13,37,58,59]. A review that gives these domains sustained attention is therefore more clinically realistic than one that treats them as background complications to a primarily respiratory disorder.

Continued attention to these domains will be essential if the field wants survival gains to be matched by gains in long-term function and health-related quality of life [13,37,58,59].

#### 4.2.2. Hepatobiliary, Sinonasal, Reproductive, and Transplant-Related Considerations

Organ-specific supportive care should extend beyond the lung and pancreas. Recent consensus recommendations have strengthened the framework for screening, evaluating, and managing hepatobiliary disease in CF, emphasizing risk-based surveillance and multidisciplinary hepatology collaboration when fibrosis or portal-hypertensive features emerge [16]. Chronic rhinosinusitis also deserves active management because sinonasal disease can worsen airway symptom burden, quality of life, and overall treatment fatigue [60].

Reproductive and family-planning issues are increasingly central in adult CF care. Males may require counseling regarding infertility and assisted reproductive options, whereas women considering pregnancy need coordinated counseling on pulmonary stability, nutritional status, medication exposure, and postpartum care [12]. These conversations should be proactive rather than deferred until conception is imminent.

Supportive care also includes planning for advanced disease. The threshold for discussion of transplant referral, symptom-directed supportive therapies, and long-term goals of care should be lower in patients with severe lung disease or accelerating decline [36]. Table 4 summarizes the principal treatment domains that remain relevant even when highly effective modulator therapy is available.

**Table 4:** Contemporary therapeutic strategies in cystic fibrosis and their practical applications.

Therapeutic Category	Representative Interventions	Primary Target or Mechanism	Typical Clinical Role
CFTR modulators	Ivacaftor; lumacaftor/ivacaftor; tezacaftor/ivacaftor; elexacaftor/tezacaftor/ivacaftor	Restoration of CFTR gating, trafficking, and surface function	Genotype-guided disease-modifying therapy; rare or non-responsive genotypes still require alternative pathways [23,24,25,26,27,28,29,30,31,32,33,34,35]
Airway clearance and mucoactive therapy	Physiotherapy, exercise, hypertonic saline, dornase alfa	Improve mucus mobilization and airway hydration	Daily foundational respiratory care, especially with persistent mucus retention or structural lung disease [8,23,36]
Anti-infective strategies	Eradication protocols, suppressive inhaled or systemic antibiotics, infection-control measures	Suppress or eliminate airway pathogens while limiting resistance pressure	Essential for Pseudomonas control, stewardship, and management of B. cepacia complex, NTM, or post-modulator infection risk [55,56,57,72]
Nutritional and pancreatic support	PERT, diet optimization, vitamin supplementation, enteral support when needed	Treat malabsorption and sustain growth	Core management of pancreatic insufficiency [8,58]
Endocrine and bone care	OGTT-based CFRD screening, insulin therapy, bone-health surveillance and treatment	Reduce systemic complications and preserve function	Required as survival and age increase, with added attention to modulator-era glucose and bone outcomes [13,37,59]
Advanced disease planning	Transplant referral, oxygen, ventilatory support in selected cases	Address progressive respiratory failure	Best integrated early rather than delayed, including timely transplant referral criteria [36]

Hepatobiliary care deserves special emphasis because liver complications may remain clinically silent until relatively advanced disease is present [16]. Consensus recommendations now support more structured approaches to screening and evaluation, but implementation varies, and many programs still face uncertainty about when to escalate from biochemical monitoring to dedicated imaging, portal-hypertension assessment, or hepatology referral. Similar gaps can arise in upper airway management, where chronic rhinosinusitis is often normalized by patients despite substantial quality-of-life impact and the possibility of sinonasal infection acting as a reservoir for lower airway organisms [60].

Reproductive and transplant-related issues further highlight the need for anticipatory rather than reactive care. Fertility counseling and pregnancy planning should begin before a patient enters a crisis or faces urgent reproductive decision-making, and transplant referral should be viewed as a component of responsible long-term planning rather than a marker of therapeutic failure [12,36]. In both settings, the quality of outcomes depends heavily on timing, shared decision-making, and close collaboration across specialties. These domains, therefore, belong inside the core architecture of CF care, not at its margins.

These organ-specific domains also illustrate why modern CF management should not be described as purely “pulmonary care plus extras.” Hepatology, otolaryngology, reproductive medicine, and transplantation medicine all contribute substantively to outcome and quality of life [12,16,36,60]. When these fields are integrated late or inconsistently, patients may face avoidable delays in diagnosis, missed counseling opportunities, or treatment plans that fail to account for cross-organ tradeoffs. A genuinely comprehensive CF review must therefore place these domains inside the mainstream of clinical management rather than in a marginal section on complications.

The same logic applies to hepatobiliary, sinonasal, and reproductive care. These domains were once sometimes treated as subsidiary to “the real disease” in the lungs, but that framing is no longer defensible [12,16,36,60]. Liver disease can change prognosis and constrain therapeutic options, chronic rhinosinusitis can impose a constant symptomatic burden and complicate microbial management, and reproductive health increasingly affects life planning for adults with CF. A mature model of care, therefore, treats these issues not as optional specialist referrals, but as predictable components of longitudinal follow-up that must be integrated into routine review, patient education, and anticipatory counseling. This perspective is particularly important in a comprehensive review because it reflects how multidisciplinary centers actually deliver modern CF care.

These considerations also reinforce the value of anticipatory counseling. Whether the issue is liver surveillance, chronic sinus management, fertility planning, or transplant referral, outcomes are generally better when conversations begin before the patient is forced into urgent decision-making [12,16,36,60]. In other words, the timing of multidisciplinary engagement is itself a therapeutic variable in CF care.

Integrating these concerns early makes the overall care plan more preventive, more realistic, and more consistent with the lifespan nature of contemporary CF medicine [12,16,36,60].

## 5. Multidisciplinary Care, Future Directions, and Conclusion

### 5.1. Lifespan Multidisciplinary Care Models

#### 5.1.1. Specialist Centers, Mental Health, and Adherence Support

High-quality CF care is inseparable from specialist multidisciplinary infrastructure. Dedicated CF centers coordinate respiratory medicine, dietetics, physiotherapy, pharmacy, microbiology, endocrinology, hepatology, psychology, and social support within a shared care model [10,45]. This structure is especially important because clinical decline in CF often reflects not a single failed intervention but the cumulative interaction of airway infection, nutrition, treatment burden, social stress, and adherence barriers.

Mental health is a particularly important example of this principle. Depression, anxiety, caregiver distress, and treatment fatigue can reduce adherence and compromise outcomes [15,68]. Routine screening and accessible referral pathways are therefore not optional quality improvements but core components of evidence-based CF care. In practice, mental-health support also helps teams navigate the shifting psychological landscape of the modulator era, where hope, uncertainty, identity change, and fear of future access problems can coexist in the same patient.

The multidisciplinary CF center model has value not only because it concentrates expertise, but because it creates continuity. Patients with CF often manage overlapping burdens in medication, airway clearance, diet, infection surveillance, glucose screening, reproductive planning, school or work demands, and emotional adaptation [10,15,45,68]. Fragmented care makes these burdens harder to prioritize and more likely to fail. A coordinated team, by contrast, can align goals, reduce conflicting recommendations, and detect subtle deterioration before it becomes clinically obvious.

Adherence support is especially critical in this context. The complexity of CF treatment means that nonadherence is rarely explained by motivation alone; it often reflects regimen burden, treatment fatigue, depressive symptoms, logistical obstacles, or competing developmental priorities [15,68]. Programs that normalize discussion of adherence, integrate mental-health review, and adapt treatment plans to lived reality are more likely to sustain long-term benefit than those that rely on repeated exhortation without structural support.

Team-based care is particularly valuable because CF management involves repeated prioritization. At any given visit, clinicians and patients may need to decide whether the most urgent issue is recurrent infection, poor weight gain, medication tolerance, school attendance, anxiety, pregnancy planning, or eligibility for a new therapy [10,12,15,45,68]. A multidisciplinary framework makes those priorities easier to balance and reduces the risk that one domain of care advances while another silently deteriorates. It also strengthens continuity when disease intensity fluctuates, which is essential in a condition defined by alternating periods of stability and abrupt clinical change.

In many ways, multidisciplinary care is the mechanism through which scientific progress becomes a real-world benefit. A new therapy can improve outcome only if patients are diagnosed in time, evaluated correctly, supported through initiation, monitored for adverse effects, and helped to integrate treatment into everyday life [10,15,45,68]. Each step requires more than a prescription. It requires communication, education, trust, and repeated reassessment. This is why specialist centers continue to matter even as some aspects of CF treatment appear pharmacologically simpler.

The center model also matters for knowledge transfer. As evidence evolves rapidly, specialist programs are better positioned to incorporate new guidance on modulators, infection control, mental-health screening, and complication surveillance in a coordinated way [10,15,45,68]. This adaptability is one of the reasons why multidisciplinary infrastructure remains essential even when specific therapies become more potent.

In this sense, multidisciplinary care is not separate from therapeutic innovation; it is the infrastructure that allows innovation to function effectively in routine life [10,15,45,68].

#### 5.1.2. Transition, Reproductive Counseling, and Long-Term Follow-Up

As more patients live into adulthood, structured transition from pediatric to adult services has become an essential component of care rather than a logistical afterthought [14,17]. Effective transition programs address health literacy, self-management, treatment ownership, reproductive counseling, insurance or access issues, and psychosocial continuity. Without such planning, gains achieved during pediatric care may be undermined by fragmented follow-up or poor therapeutic engagement in early adulthood.

**Figure 2:**
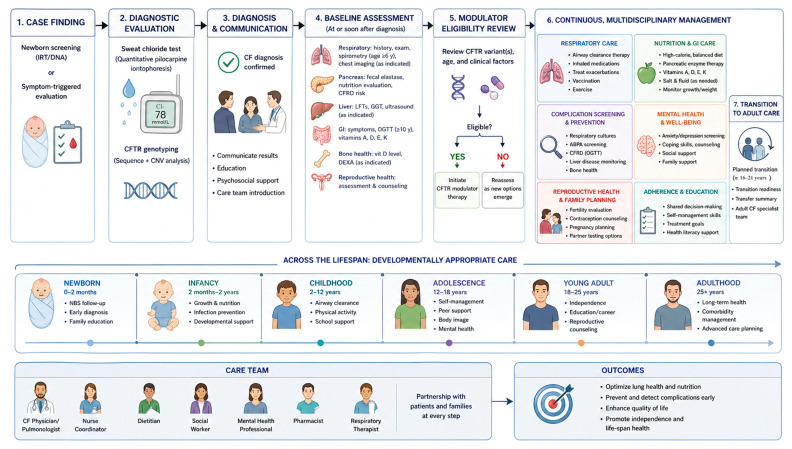
Genomics-informed diagnostic and multidisciplinary management pathway for cystic fibrosis across the lifespan. Legend: The pathway begins with newborn screening or symptom-triggered evaluation, proceeds through sweat chloride testing and CFTR variant analysis, and then links diagnosis to baseline organ assessment, review of therapeutic eligibility, respiratory and nutritional management, complication screening, mental-health support, reproductive counseling, and transition to adult specialist care. The figure is intended as a conceptual care framework rather than a quantitative genomic workflow diagram.

Figure 2 depicts a genomics-informed lifespan care pathway that links screening and diagnosis to variant interpretation, modulator review, routine surveillance, psychosocial care, and transition planning. The broader lesson is that precision medicine alone does not guarantee durable benefit; its success depends on systems capable of interpreting genomic data and then delivering, monitoring, and sustaining complex long-term therapy [9,10,11,12,14,15,17].

Transition in CF is best understood as a developmental process rather than a transfer event [14,17]. Adolescents and young adults must gradually assume responsibility for treatment routines, appointment management, symptom recognition, and health-system navigation, all while negotiating education, work, relationships, and evolving identity. Because this period often coincides with changes in adherence and psychosocial stress, transition planning should be staged, repeated, and individualized rather than reduced to a one-time checklist.

Long-term follow-up also needs to account for the fact that expectations are changing. Patients who once anticipated a short life may now confront decisions about higher education, parenting, employment, aging, and late complications in ways that previous generations of CF care did not routinely address [10,12,14,17]. This shift requires clinicians to integrate prognosis, reproductive counseling, health maintenance, and psychosocial planning into routine follow-up, even for patients who appear clinically stable.

Long-term follow-up in CF increasingly resembles chronic disease management in other adult specialties, but with a unique inherited-disease overlay. Questions about employment, travel, intimacy, parenting, insurance, and future health planning now arise more often because survival has improved [10,12,14,17]. This creates a new responsibility for CF teams: they must be prepared not only to manage disease, but to support patients living fuller and longer lives with that disease. The maturity of a modern CF program can therefore be judged partly by how well it handles these longitudinal life-course issues.

Structured transition and long-term follow-up also serve an epistemic function: they allow the field to observe how CF is changing in real time. Earlier diagnosis, better supportive care, and more effective pharmacotherapy are generating new patient trajectories that differ meaningfully from those described in older literature [10,12,14,17,32,33]. Adult services, therefore, need to be prepared not just to inherit historically established diseases but to manage emerging phenotypes shaped by earlier intervention and longer survival. Registries, coordinated transition pathways, and sustained adult specialist review are essential if the field is to learn from that changing landscape rather than remain anchored to outdated assumptions.

A strong transition pathway is therefore not only a service design preference but a mechanism for preserving therapeutic gains achieved in childhood [14,17]. As more individuals with CF reach adulthood in better health than previous generations, continuity of care will increasingly determine whether early-life advances translate into durable adult outcomes.

As outcomes improve, these longitudinal care structures will become even more important rather than less, because the demands of adult-life planning will continue to expand [12,14,17].

### 5.2. Emerging Therapeutics, Access Challenges, and Clinical Outlook

#### 5.2.1. Mutation-Agnostic and Next-Generation Therapies

Future CF therapy will likely combine improved modulators with mutation-agnostic platforms such as messenger RNA replacement, gene addition, gene editing, and cell-based repair [20,40,47]. These approaches are most relevant for patients with nonsense, splice, or other rare variants that remain incompletely served by current pharmacologic options.

The main barriers remain airway delivery, durability, immune response, manufacturing scale, and the choice of clinically meaningful outcome measures [20,47]. Patient-derived organoids and related functional rescue assays are increasingly useful for theratyping and for prioritizing therapy in rare-variant settings where conventional trials are difficult to perform [20,40,47,53].

Future therapeutic development will also require better integration between laboratory models and clinical decision-making. Patient-derived intestinal and airway organoids, together with other functional rescue assays, are increasingly used to estimate individual drug responsiveness, especially for rare variants that cannot be studied easily in large conventional trials [20,40,47,53]. As the field becomes more individualized, the evidence structure for approving or selecting therapy may need to diversify, combining classic randomized trials with high-quality translational and real-world response data. This evolution would be particularly valuable for patients whose genotypes are too rare to fit traditional large-trial frameworks.

The most likely future is therefore additive rather than substitutive: modulators, supportive care, and selected upstream correction strategies will probably coexist within the same long-term treatment framework [20,40,47].

#### 5.2.2. Equity, Implementation, and the Future of CF Care

A major unresolved issue in CF is inequity. Therapeutic innovation has progressed faster than global access, leaving major disparities in diagnosis, treatment availability, and long-term outcomes across regions and healthcare systems [10,32,33].

The clinical future of CF therefore depends on two linked goals: developing safer and broader therapies, and ensuring that existing gains in screening, specialist care, infection control, nutritional support, mental-health care, and modulator treatment are implemented equitably over time [8,9,10,11,15,47].

For that reason, the future of CF care should be framed not only as drug discovery, but also as the coordinated improvement of diagnosis, access, multidisciplinary care, and longitudinal outcome monitoring [8,9,10,11,15,47].

Meaningful progress will ultimately be judged by the breadth of benefit: earlier diagnosis, fewer treatment disparities, better preserved organ function, and more stable long-term quality of life for patients and families [8,9,10,11,15,32,33,47].

## 6. Conclusions

Cystic fibrosis has evolved from a uniformly life-shortening pediatric disorder into a chronic multisystem disease increasingly managed across the lifespan. This change reflects advances in molecular genetics, newborn screening, specialized multidisciplinary care, and CFTR modulators. Contemporary understanding of CF now extends beyond defective chloride transport alone and includes bicarbonate secretion, mucus dehydration, impaired mucociliary clearance, chronic infection, inflammation, and progressive structural tissue injury. At the same time, the genomics of CF is no longer reducible to a short list of canonical variants; clinically meaningful interpretation increasingly depends on full-gene analysis, rare-variant curation, splice-aware evaluation, and integration of genotype with functional and longitudinal phenotypic data.

Important challenges remain. Current modulator therapy has transformed outcomes for many patients, but it does not fully reverse established organ damage and does not adequately serve all CFTR genotypes. Future progress will depend on more precise diagnostic interpretation, sustained multidisciplinary follow-up, broader access to care, and continued development of mutation-agnostic strategies such as RNA-, gene-, and cell-based approaches.

## Data Availability

No new data were created during this study.

## Abbreviations

The following abbreviations are used in this manuscript:

CF	Cystic fibrosis
CFTR	cystic fibrosis transmembrane conductance regulator
CFRD	Cystic Fibrosis-Related Diabetes
